# Hyderabad Ocular Morbidity in Elderly Study (HOMES) – Rationale, Study Design and Methodology

**DOI:** 10.1080/09286586.2019.1683867

**Published:** 2019-10-28

**Authors:** Srinivas Marmamula, Navya Rekha Barrenkala, Rajesh Challa, Thirupathi Reddy K, Shashank Yellapragada, Satya Brahmanandam M, David S. Friedman, Rohit C. Khanna

**Affiliations:** a Allen Foster Community Eye Health Research Centre, Gullapalli Pratibha Rao International Centre for Advancement of Rural Eye care, L V Prasad Eye Institute, Hyderabad, India; b Brien Holden Institute of Optometry and Vision Science, L V Prasad Eye Institute, Hyderabad, India; c Wellcome Trust/Department of Biotechnology India Alliance, L V Prasad Eye Institute, Hyderabad, India; d School of Optometry and Vision Science, University of New South Wales, Sydney, Australia; e Dana Center for Preventive Ophthalmology, Wilmer Eye Institute, Johns Hopkins University School of Medicine, Baltimore, Maryland, USA

**Keywords:** Visual impairment, elderly, residential care, refractive errors, India, HOMES

## Abstract

***Purpose***: To describe the study design, interobserver variability of the questionnaires and clinical procedures of Hyderabad Ocular Morbidity in Elderly Study (HOMES) designed to, (a) to investigate the prevalence, causes and risk factors for visual impairment, and (b) to assess the impact of dispensing spectacles and cataract surgery on visual functions, fear of falls (FOF) and depression among the elderly in India.

***Methods***: Individuals aged ≥60 years are considered elderly. The non-clinical protocol was administered by two trained investigators and included collection of personal, sociodemographic information, ocular and systemic history, Indian Visual Function Questionnaire (IND-VFQ33), Patient Health Questionnaire (PHQ9), Mini-Mental State Examination (MMSE) questionnaire, Hearing Handicap Inventory for the Elderly Screening (HHIE), Short Falls Efficacy Scale (SFES) questionnaire. The eye examination was conducted by a trained optometrist and vision technicians in clinics set-up in the homes and included visual acuity (VA) assessment for distance and near, anterior segment examination and fundus examination, and imaging. The reliability assessments were carried out among 138 participants.

***Result***: The intraclass correlation (ICC) coefficients for MMSE, PHQ9, HHIE, SFES was 0.73 (95% CI: 0.62–0.81), 0.67 (95% CI: 0.54–0.77), 0.63 (95% CI: 0.48–0.74) and 0.70 (95% CI: 0.58–0.79) respectively. The ICC for INDVFQ domains ranged from 0.66 (95% CI: 0.55–0.74) for Psychosocial Impact to 0.88 (95% CI: 0.84–0.91) for activity limitation. The ICC for VA was 0.94 (95% CI: 0.92–0.96).

***Conclusion***: All questionnaires demonstrated acceptable reliability and can be applied in the main study. HOMES is expected to provide data that will help plan strategies to contribute towards ‘healthy aging’ in India.

## Introduction

Globally, 253 million people are visually impaired, of which about 85% of them are 50 years of age and older.^1^ India is home to 8.8 million blind individuals and nearly 50 million people with moderate-to-severe visual impairment (MSVI).^1^ Over 75% of all visual impairment is avoidable (preventable or treatable). Both cataract and uncorrected refractive errors remain the leading causes of blindness, and uncorrected refractive errors remain the leading cause of SVI.^2^ While cataract surgery is one of the most cost-effective interventions in healthcare,^3,4^ uncorrected refractive errors can be corrected using spectacles.

Visual impairment is more common among the elderly living in residential care when compared to those living in their own homes in the communities and non-institutionalized environments.^5–8^ A significant proportion of this visual impairment among the elderly in residential care can be corrected by simple interventions such as spectacles and cataract surgery.^9–12^ Studies have shown that visual impairment in the elderly affects all dimensions of their life including mobility, self-care, driving, participation in social and religious activities and overall quality of life.^13–15^ Visual impairment is also associated with an increased risk of mortality.^16–18^ Reports have shown a delay in the risk of mortality as a result of cataract surgery,^19,20^ and correction of refractive errors resulting in a significant improvement in the quality of life.^21,22^ Furthermore, visual impairment may lead to falls which may lead to fractures resulting in an adverse impact on the quality of life of the individuals.^23–28^

Even though the phenomenon of ‘homes for the aged’ centres is a recent concept in India, these are a well-organized sector and have been in existence for several decades in developed countries.^29^ In India, the home for the aged centres are diverse both in terms of scope, amenities provided and the number of elderly living in each centre. These homes are typically run by non-governmental, religious or voluntary organizations with support from the government and philanthropists. In some homes, either the elderly person or their kin pay the ‘user fee’. Most of these homes offer food and accommodation, and private homes are even staffed with nursing staff to attend the medical needs and other support staff to assist elderly residents in daily routine tasks.^29^

Sound epidemiological data on prevalence and causes of visual impairment are needed to formulate strategies to address vision loss in the elderly in residential care. Research on the prevalence and impact of visual impairment among the elderly population has been carried out in developed countries but such research is still in a stage of infancy in India.^5,30–35^ To address this lacuna, a well-designed epidemiological study focused on the elderly is needed. In this background, The Hyderabad Ocular Morbidity in Elderly Study (HOMES) is being undertaken. HOMES is a longitudinal study designed (A) to investigate the prevalence, causes and risk factors for visual impairment among elderly individuals living in residential care facilities in Hyderabad, India, (B) to assess the impact of interventions such as spectacles and cataract surgery on visual functions in elderly living in residential care, and (C) to assess the prevalence of fear of falls (FOF), depression and hearing impairment, and their association with vision loss. This paper describes the study design and methodology adopted for HOMES.

## Materials and methods

### Ethics approval

The study protocol was approved by the institutional review board of the Hyderabad Eye Research Foundation, L V Prasad Eye Institute, Hyderabad, India. The study is currently being carried out in accordance with the Declaration of Helsinki. The elderly residents are enrolled in the study after obtaining written informed consent. Approval from the administrative head of the home for the aged centre is also being obtained before starting the data collection process.

### Study design and sampling method

**Figure 1.**
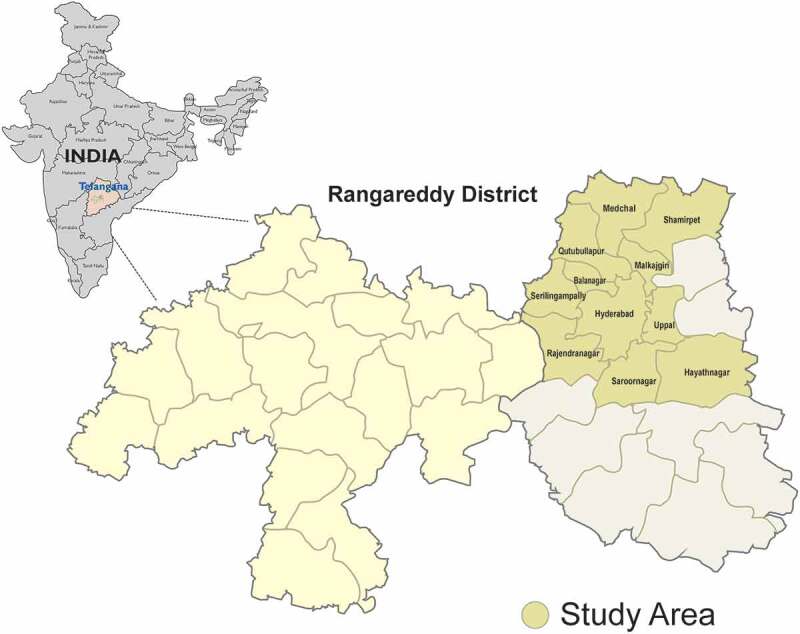
The map showing the study location.

HOMES is a longitudinal study with pre and post-intervention phases and is being carried out in Hyderabad, a city in the south Indian state of Telangana and in its surrounding regions (Figure 1).

For the purpose of the study, each ‘home for the aged’ centre is considered as a cluster. Based on an anticipated prevalence of avoidable visual impairment of 20%, a precision in the estimate of 20% of the prevalence, a non-response rate of 25%, a design effect of 1.4 to account for clustering, a sample size of 666 individuals is required. The prevalence estimates were based on our previous publication on visual impairment in the elderly in residential care.^34^ We anticipate a lower prevalence in HOMES compared to our previous study which was done in rural areas whereas HOMES is carried out in urban locations.^34^ Table 1 shows the sample sizes for a cluster size of 40, 50 and 60 individuals with varying anticipated prevalence. For logistical reasons, only those homes with at least 10 freely mobile residents are included in the study.

**Table 1.** Sample size calculations for various cluster sizes, expected prevalence of visual impaiment (VI) and number of people with VI in the sample.

Expected Prevalence of VI	Cluster size	Design effect	Sample size with 25% inflation	Expected number of homes to be covered for the estimated sample	Expected number of participants with VI
15%	40	1.4	916	23	137
15%	50	1.5	982	20	147
15%	60	1.6	1047	18	157
20%	40	1.4	666	17	133
20%	50	1.5	714	15	143
20%	60	1.6	761	13	152
25%	40	1.4	509	13	127
25%	50	1.5	545	11	136
25%	60	1.6	581	10	145

A list of homes was obtained from HelpAge India, a non-governmental organization working for the aged care and also from other resources such as governmental publications. There are 110 homes for the aged in this region. The number of residents in these homes ranges from 10 to over 80 with most institutions having about 40 to 50 residents. All the homes within 50-km radius of the L V Prasad Eye Institute are mapped and the required number of homes is randomly selected for the study. The residents aged ≥60 years at the time of enumeration and residing in these homes for at least a period of one month and agreed to participate are included in the study.

### Definitions

Visual impairment is defined as presenting visual acuity worse than 6/18 in the better eye and included blindness (worse than 3/60), severe visual impairment (worse than 6/60 to 3/60) and moderate visual impairment (worse than 6/18 to 6/60). Visual impairment caused due to cataract and uncorrected refractive errors will be considered as avoidable visual impairment. Refractive error is defined as spherical equivalent (i.e. sphere + ½ cylinder) > ± 0.50 Diopter in the better eye and the functional definition of refractive error are defined as the change in at least one visual impairment category with the best refractive correction. Cataract is defined as the presence of any lenticular opacity that causes visual impairment. Presbyopia is deemed to be present if a presenting near vision is worse than N8 but improves to N8 or better with near correction.

The main cause of visual impairment is assigned by the clinician first for the eye and then for the person. In cases where there are multiple causes, based on the clinical examination and the retinal images, the cause that is more likely to explain the vision loss is considered as the main cause in that eye. At the person level, in cases, where there are different causes of visual impairment in both the eyes, the cause that is more easily correctable or treatable is given precedence over other causes. For example, if cataract is the cause for visual impairment in the right eye and uncorrected refractive error in the left eye, then at the person level, uncorrected refractive error is marked as the main cause and used for analysis.

### Study team

The study team comprises two social investigators (professionals with a master’s degree in social work and trained is survey research methods). The clinical team includes an optometrist and vision technicians (personnel trained to provide primary eye care including basic eye examination, visual acuity assessment and refraction) who visit the home for the aged centres and conduct eye examinations. The logistics for the study is organized by a study coordinator, who also takes care of the referrals. At the data centre, two data entry personnel are involved in data entry. The study is led by the principal investigator who has overall responsibility for the scientific and administrative aspects of the study.

### Non-clinical protocol

The non-clinical protocol included the administration of questionnaires by the trained investigators.

The questionnaires included personal, sociodemographic, ocular and systemic history, Indian Visual Function Questionnaire (IND-VFQ 33),^36,37^ Patient Health Questionnaire (PHQ-9),^38^ Mini-Mental State Examination (MMSE) questionnaire,^39^ Hearing Handicap Inventory for the Elderly Screening (HHIE-S),^40^ Short Falls Efficacy Scale (SFES) questionnaire.^41,42^ Based on interviewers' observations, mobility status of the participants is classified as ‘independently mobile’, ‘mobile with assistance’ and ‘immobile/bedridden’.

Independent bilingual reviewers translated all the questionnaires from English language into the local language (Telugu) and also reverse translated into English to ensure that the content of the questionnaire remain unchanged. The questionnaire administration process is audio recorded for random reviews as a part of the quality control measure. A folder containing all the study instruments (forms) is prepared for each participant by the investigators who conduct the interviews in the homes. The consent form in the local language (Telugu) is also available in the folder. Once the interviews are completed, the folders are verified and handed over to the study coordinator who will, in turn, pass it on to the clinical team for an examination.

Participants who were bedridden are examined but not all questionnaires are administered. This shorter version of assessment included personal and demographic information, systemic and ocular history, Washington disability questionnaire and clinical examination. The interviews were attempted on all the independently mobile individuals. After the collection of personal and demographic information, mini-mental state examination (MMSE) assessment questionnaire is administered. If the MMSE score is less than 20 (suggestive of mild cognitive impairment), then questionnaires were restricted to systemic and ocular history, risk factors and complete clinical examinations. For all those individuals whose MMSE scores are greater than or equal to 20, the remaining set of questionnaires were administered, and complete examination protocol is carried out as shown in Table 2.

**Table 2.** Study questionnaires used in the HOMES project.

Code	Instrument name	Details included	Grading / Comment
1A	Home for the Elderly profile	Details of the home, address, type of home, funding source, amenities	Descriptive data
1B	Personal and socio demographic information	Age, gender, education, previous occupation, family structure	Based on the age, the participants are classified as young old (60–69 years), Middle old (70–79 years) and older old (80 years and older).
2	Cognition Function	Mini Mental State Examination questionnaire is used to assess cognition function (19 Questions)	<24 is considered as abnormal. No cognitive impairment = 24–30; mild cognitive impairment = 19–23; moderate cognitive impairment = 10–18; and severe cognitive impairment≤9.
3	Risk factors	Smoking, alcohol consumption, both current and the past use	Binary variable; yes/no
4	Visual Functions	Rasch analyzed Indian Visual Function Questionnaire (IND-VFQ 33) is used. It included 33 questions covering four domains (General functioning (21), psychosocial impact (5), Visual symptoms (7))	Sum of scores of each of the domains/Maximum score possible for each domain
5	Depression	Patient Health Questionnaire (PHQ 9) is used to assess depression	PHQ-9 scores of 5, 10, 15, and 20 represented mild, moderate, moderately severe, and severe depression, respectively
6	Hearing	Hearing is assessed using (10 questions) Hearing Handicap Inventory for the Elderly Screening questionnaire	0–8 suggest no hearing handicap, 10–24 suggest mild-moderate hearing handicap, 26–40 suggest significant hearing handicap
7	Fear of Falling and falls history	Falls history is assessed. Fear of falling is assessed using Short Falls Efficacy Scale (SFES) questionnaire	Fear of Falling is graded as : Low Concern: 7–8, Moderate Concern: 9–13, and High Concern: 14–28
8	Performed based measures	Included computer based tests such as face recognition, facial expression recognition, visual search tasks	Accuracy and time taken to accomplish the task
9	Systemic history	History of systemic conditions such as hypertension, diabetes, heart disease and others are asked along with duration and current medication	Binary variable; yes/no Deatils of current medication
10	Ocular history	Included cataract surgery, spectacles use, and other eye care services obtained	Binary variable; yes/no Details of the services obtained
11	Measures of frailty and anthropometry	Short Physical Performance Battery (SPPB)^42^ (Gait speed test (3-meter distance), Chair stand test, Hand grip strength), Height, weight, blood pressure	Continuous / Ordinal scale
12	Clinical Examination	Visual acuity assessment, refraction, slit lamp examination, applanation tonometry, fundus examination and retinal imaging	World Health Organization Categories of visual impairment

The questionnaire administration is always done before the clinical examination. After the administration of questionnaires, anthropometry measures and performance-based measures (PBM)^43^ are carried out by the trained vision technician. This is followed by clinical eye examination by trained optometrist and vision technicians. Clinical competence of vision technicians in conducting the clinical examinations are published.^44,45^ The interviews and non-clinical examination are always scheduled on different days to ensure that elderly participants are not tired and ample time is provided to take rest in between the procedures if necessary.

### Clinical examination protocol

The clinical examination protocol includes visual acuity assessment, stereopsis, near-vision assessment, refraction, slit-lamp biomicroscopy and fundus examination. *Distance* visual acuity (VA) is measured with the standard log MAR (logarithm of the minimum angle of resolution) chart with tumbling E and English alphabet optotypes under good illumination of at least 180 lux measured using a light meter.The VA is tested with the subject’s current refractive correction if glasses are being used. Apart from this, unaided VA and pinhole VA is recorded. *Near* VA is measured at a distance of 40 cm using the log MAR near vision “E” chart with the current refractive correction, if any, along with unaided acuity. For VA measurement with log MAR chart, letter by letter scoring method is used for distance and near vision. For participants uncooperative for VA assessment using the log MAR chart, fixation pattern, steadiness, and maintenance of fixation are assessed using a torchlight. Reverse contrast VA (white optotypes with a black ground) is also measured. *Contrast sensitivity* is measured with the MARS contrast sensitivity chart (Mars Perceptrix Corporation (Chappaqua, NY)). It consists of 48 letters of 1.75 cm height arranged in eight rows of six letters each. The contrast of the optotypes ranges from 91% (–0.04 log units) to 1.2% (1.92 log units) with the contrast of each letter decreasing by a constant factor of 0.04 log units. Each letter subtends 2° at the test distance of 50 cm (equivalent to 20/480). This test is conducted using habitual spectacle correction. Numerical optotypes are used in this study.

*The external examination*, including ocular motility using broad H test and cover tests, is done on all subjects. Ocular alignment is assessed by the Hirschberg test using a pen torch light with the refractive correction being used by the subject. *Refraction* is done for all subjects who have presenting distance worse than 6/12 or near vision worse than N8 in either eye. Both manual and autorefraction and acceptance are also done. Best-corrected distance and near VA are assessed and documented. *Slit lamp Examination* is conducted using a portable slit lamp (BA 904 Haag-Streit Clement Clarke International, UK). The eyelids, conjunctiva, cornea, anterior chamber, iris/pupil and lens are examined. Intraocular pressure (IOP) is measured using hand-held Perkins applanation tonometer (Mk3 Haag-Streit Clement Clarke International, UK) after administration of topical anesthetic eye drops and fluorescein stain. The probe of the tonometer is cleaned with alcohol swabs after examining each participant. Intraocular pressure is estimated digitally for those who are uncooperative with tonometry or have an active infection. *Fundus examination and retinal photography* is done using a non-mydriatic fundus camera (Zeiss Visuscout). Images are reviewed by experts and are graded.

### Intervention for visual impairment

Participants with visual impairment in one or both eyes are referred to L V Prasad Eye Institute for a comprehensive eye examination. All services including eye examinations, surgeries, ophthalmic lasers and spectacles are provided at ‘no-cost’ to the participants. Clinical consultations at the institute are facilitated and transportation assistance is provided wherever required. The clinical protocol and the questionnaires are administered twice to all participants, once at the baseline and then again after 3–6 months after the intervention for cataract, refractive errors and other conditions. Only one complete assessment is done after the intervention.

### Reliability study

A reliability study was set up to assess the inter-examiner reliability of the questionnaires and the clinical measurements. The inter-examiner reliability assessment was done between the two examiners for Mini-Mental State Examination (MMSE), Indian Visual Function (IND-VFQ) Questionnaire, Patient Health Questionnaire (PHQ-9), Hearing Handicap Inventory (HHIE), Short Falls Efficacy Scale (SFES) questionnaires. Intraclass Correlation Coefficient (ICC) was used to assess inter-examiner reliability.^46^ ICC estimates and their 95% confident intervals were calculated using Stata Statistical Software version 14 based on a mean-rating (*k* = 2), consistency definition, single rater type, two-way mixed-effects model. For visual acuity assessment, a senior optometrist was used as the gold standard. All instruments have shown acceptable reliability of at least 0.6 or more (Table 3). After the reliability study, a pilot study was done in four home for the aged centres with an aim to understand the workflow dynamics and to fine-tune the protocols for the main study.

**Table 3.** Results of the reliability study.

Questionnaire/Procedure	n	Intra Class Correlation quotient (ICC) (95% CI)
Mini Mental State Examination (MMSE)	92	0.73 (0.62–0.81)
Indian Visual Function Questionnaire (IND VFQ) domains		
Mobility	138	0.84 (0.78–0.88)
Activity limitation	138	0.88 (0.84–0.91)
Psychosocial impact	138	0.66 (0.55–0.74)
Visual symptoms	138	0.83 (0.76–0.87)
Patient Health Questionnaire (PHQ9)	92	0.67 (0.54–0.77)
Hearing Handicap Inventory (HHIE)	92	0.63 (0.48–0.74)
Short Falls Efficacy Scale (SFES) questionnaire	89	0.70 (0.58–0.79)
Visual acuity assessment	101	0.94 (0.92–0.96)

### Pilot study

**Figure 2.**
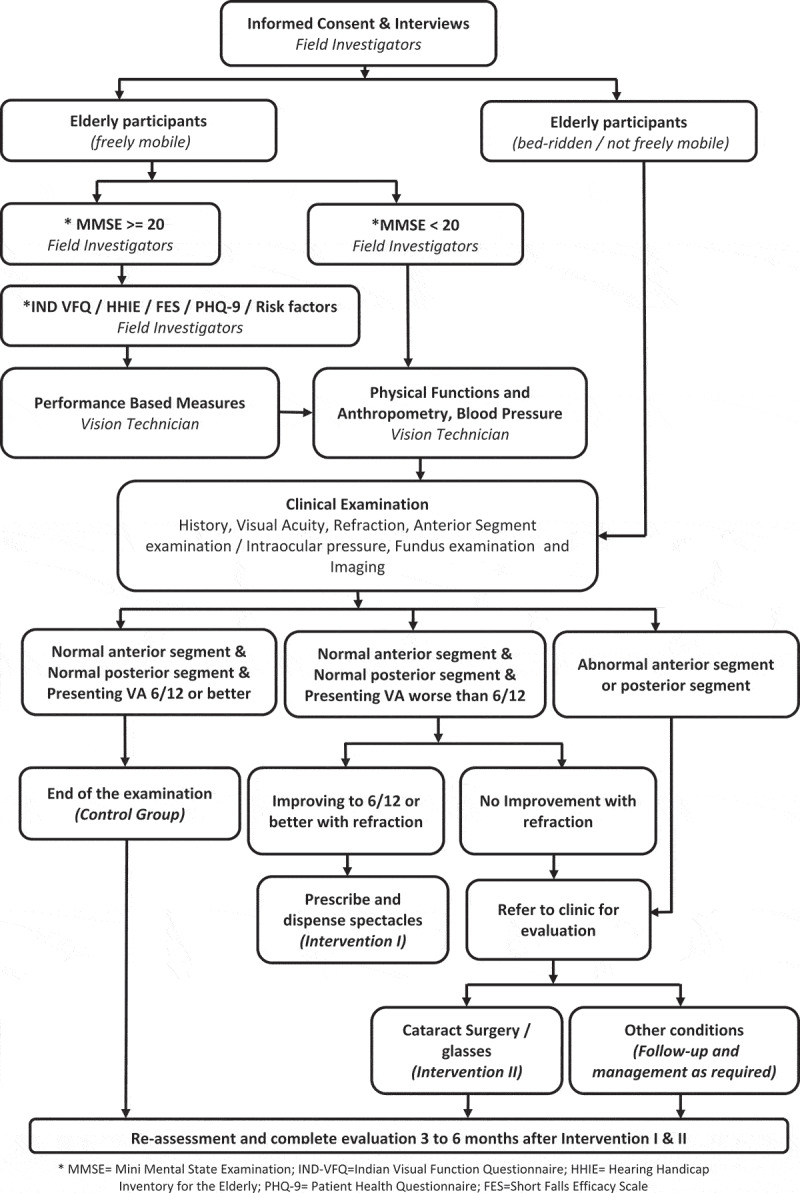
Flowchart showing the sequence of eye examination.

In total, 64 elderly participants were examined from four home for the aged centres. The mean age of these participants was 76.7 years (standard deviation of 9.3 years; range 60 to 99 years). Among those examined, 50% were women. In total, 59 (92.2%) of the participants were freely mobile and 5 (7.8%) were bed-ridden. Based on presenting visual acuity, visual impairment was present in 28.1% (95% CI: 17.6–40.8) of the participants which decreased to 17.2% (95% CI: 8.9–28.7) with the best correction suggestive of a large unmet need for spectacles. Of the 50 participants to whom questionnaires were administered, 18% had moderate to severe depression on PHQ-9 questionnaire, 50% had moderate to high concern of falls on Short Falls Efficacy Scale (SFES) questionnaire, 26% reported mild to significant hearing impairment on the Hearing Handicap Inventory for Elderly (HHIE) questionnaire. A ‘make-shift’ clinic was made in each of the homes and all the study instruments were administered successfully. Based on the outcomes of the pilot study, the main study is being conducted. The flow chart of the examination protocol finalized for the main study is shown in Figure 2.

### Data management and analysis plan

Data on each participant are recorded on HOMES data collection forms. After interviews and clinical examination, all the completed data folders are received at the data centre and entered the database developed in Microsoft Access® with built-in validation checks for minimizing data entry errors using double data entry. The data are backed up daily and stored securely in a network server. The hard copies of the folders are stored in an iron cupboard in termite-free environment in the data centre under lock and key accessible only to the data entry personnel and principal investigator. Data analysis is conducted using Stata Statistical Software for Windows, version 14 (StataCorp. 2015. *Stata Statistical Software: Release 14*. College Station, TX: StataCorp LP). The statistical tools used for data analysis include prevalence estimates with their confidence intervals, chi-square and t-tests for univariate analyses, and logistic regression for multivariate analyses. The regression models will be assessed using variance inflation factors (VIF) and goodness of fit tests. The digital images and audio files of the interviews are systematically archived using the participant identification numbers which is a unique number allotted to every participant and this number common across all the databases.

#### Prevalence and causes of visual impairment

The prevalence and causes of visual impairment will be calculated and presented with 95% confidence intervals. Multivariable analysis will be conducted using binary logistic regression analysis to assess the association between visual impairment and age, gender, education, systemic conditions such as diabetes, hypertension and other risk factors.

#### Impact of interventions to improve vision

The Indian Visual Function questionnaire scores of those with and without visual impairment will be compared at the baseline. Post-intervention, re-assessment and comparisons of the visual function scores will be made between baseline and post-intervention for each participant. The baseline performance-based measure scores will be compared with the post-intervention scores.

#### Falls, fear of falling and visual impairment

The prevalence of falls and fear of falling will be estimated and presented with 95% confidence intervals. Multivariable analysis will be conducted using binary logistic regression analysis to assess the association between falls, fear of falls and visual impairment, age, gender, education, systemic conditions such as diabetes, hypertension, hearing, cognition and frailty measures.

## Discussion

The HOMES study is one of the most comprehensive eye health studies being done on the elderly age group living in residential care in India. This study also aims to understand factors such as cognition, depression, hearing, falls and their association with vision loss. At this juncture when India is experiencing a demographic shift with an increasing proportion of the elderly population, the results from this study can provide valuable insights to develop long-term comprehensive geriatric health strategies including eye care services in India.

The methodology and the clinical procedures adopted in this study are based on several published studies. The study protocols are adapted to suit the Indian context and local applicability. For example, the Indian Visual Function questionnaire is country-specific and the use of this questionnaire in this study is expected to provide valuable insights on visual functions and change in visual functions due to intervention. The performance-based measures are modified to suit the local context. All the protocols were successfully field-tested in the pilot study and necessary modifications were done before applying in the main study.

Though the limitation of generalizability of the reulst of this study to the general population at large remains as the study is restricted to home for the aged centres, it is expected to provide useful insights on the impact of interventions for vision loss in the elderly in India. As interviews of elderly who are bed-ridden are restricted to socio-demographic information only, this will remain a limitation as the reason for illness due to which the elderly have become bed-ridden is not readily available.

In conclusion, the HOMES study is one of the most comprehensive eye health studies on the elderly age group living in residential care in India. The pilot study has provided valuable insights on several factors such as clinic setup and time duration for each eye examination for the main study. A clear workflow was established for various study procedures. It also provided information for planning logistics for the main study. The data collection is expected to take two years. The information from this study is expected to contribute significantly to the development of strategies to achieve the overarching goal of ‘healthy aging’ and ‘happy aging’ in India in the years to come.
